# Supplementary material for: Disparities in Oral Nutritional Supplement Usage and Dispensing Patterns across Primary Care in Ireland: ONSPres Project
Source: Nutrients. 2022 Jan 14;14(2):338. doi: 10.3390/nu14020338 (PMC8781404; doi:10.3390/nu14020338)
Supplement: Supplementary file 1 [file nutrients-14-00338-s001.zip › nutrients-1517266-supplementary.pdf]

Table S1: Category and nutritional content of oral nutritional supplements

ONS Category	Range of energy (kcal/mL)	Range of protein (g) per serving	% of protein energy
Very high energy sip feeds[a]	2.0-2.4	11.8-20.2	16-20
High energy, standard protein sip feeds[a, b]	1.5-1.9	10.6-20.0	3-24
High energy modular[c]	3.3-5.0	0.0-8.0	0-8
High protein sip feeds	1.25-1.6	15.8-20.0	24-32
High energy semi-solids	1.4-2.0	7.1-12.5	17-24
Texture-modified high energy high protein sip feeds	1.5-2.4	12.0-20.0	16-27

ONS: Oral nutritional supplement. [a]includes ONS with or without added fibre, [b]includes milk or juice-based products and powdered milkshakes that contain 1.9 kcal/mL, [c]includes fat and/or carbohydrate formulations in shot or sachet form.

Nutritional composition obtained from manufacturers' data.
